# L22 ribosomal protein is involved in dynamin-related protein 1-mediated gastric carcinoma progression

**DOI:** 10.1080/21655979.2022.2045842

**Published:** 2022-03-01

**Authors:** Jianghong Cheng, Zizhuo Sha, Ruisan Zhang, Jinghao Ge, Peng Chen, Xuefeng Kuang, Jiazhi Chang, Kai Ren, Xianyang Luo, Shuai Chen, Xingchun Gou

**Affiliations:** a Shaanxi Key Laboratory of Brain Disorders and School of Basic Medical Science, Xi’an Medical University, Xi’an, China; b Department of Otolaryngology-Head and Neck Surgery, The First Affiliated Hospital of Xiamen University, Xiamen, China; c Institute of Basic and Translational Medicine, Xi’an Medical University, Xi’an, China; d Xiamen Key Laboratory of Otolaryngology Head and Neck Surgery, Xiamen, China

## Abstract

Mitochondrial fission depends on dynamin-related protein 1 (Drp1) guanosine triphosphatase activity. Although there is some association between Drp1 and gastric cancer, the detailed mechanism remains largely unknown. In this study, the elevation of Drp1 was observed in human gastric carcinoma specimens including gastric mixed adenocarcinoma tissues, gastric intestinal-type adenocarcinoma tissues, and human gastric cancer cells compared to normal control, but not in diffuse gastric adenocarcinoma tissues. Gastric cancer patients with high Drp1 harbored advanced pathological stages and poor progression-free survival probability compared to those with low Drp1. Mdivi-1-mediated inactivation of Drp1 robustly inhibited cell viability and tumor growth but conversely induced cell apoptotic events *in vitro* and *in vivo*. Based on the Encyclopedia of RNA Interactomes Starbase, L22 ribosomal protein (RPL22) was recognized as the potential downstream oncogene of Drp1. Clinically, the significant correlation of Drp1 and RPL22 was also verified. Mechanistically, Drp1 inactivation did not affect the accumulation of RPL22 in gastric carcinoma. However, the intracellular distribution of RPL22 had an endonuclear location in Drp1-inactivated tumors. Of note, Drp1 inactivation notably reduced the expression of cytoplasmic RPL22 and increased its nuclear level in gastric cancer cells. Collectively, Drp1 had high levels in human gastric carcinoma specimens and could serve as a potential diagnostic and prognostic biomarker in gastric carcinoma. The Drp1 inactivation-mediated anti-proliferative and pro-apoptosis effects on gastric cancer were possibly associated with nuclear import of RPL22. This knowledge may provide new therapeutic tools for treating gastric carcinoma via targeting mitochondria-related ribosome pathway.

## Introduction

Gastric cancer is the fifth most common cancer and the third leading cause of cancer-related death worldwide [1]. More than 1 million new cases of gastric cancer have been diagnosed worldwide in 2018 [2]. Helicobacter pylori (H. pylori) infection and dietary risk factors such as nitrites and salted foods are the main causes of gastric cancer [3]. Although the improved surveillance approaches in the diagnosis and treatment strategies have been developed, the molecular pathogenesis of gastric carcinoma has not been adequately investigated. Accumulating evidence indicates a critical role of mitochondria dysregulation in tumorigenesis. Mitochondrial fission exhibits a decisive step in regulating mitochondrial integrity and cancer cell viability via induction of excessive production of reactive oxygen species and ATP production [4]. Alterations in mitochondrial fission dynamics contribute to the maintenance of bioenergetic homeostasis and cellular health, and subsequently the control on gastric cancer development.

As the main regulator in mitochondrial fission process, dynamin-related protein 1 (Drp1), encoded by *DNM1L*, is a member of the dynamin family of guanosine triphosphatases (GTPases), playing a vital role in multicellular processes such as cell proliferation, differentiation, apoptosis, and metabolism [5]. Drp1 participates in the numerous physiological and pathological process in mammals and regulates multiple human diseases through mitochondrial dynamics [6]. Of note, a growing body of evidence suggests that Drp1-mediated mitochondrial fission is closely associated with tumorigenesis such as hepatocellular carcinoma, colon cancer, lung cancer, and glioblastoma, acting as a promising therapeutic target for treating cancer [^7–9^]. Drp1 regulates the progression and occurrence of most cancer through a variety of functional mechanisms, including the regulation of cell proliferation and apoptosis, the alterations of mitochondrial energetics and cellular metabolism dynamics, and the promotion of invasion and metastases [10]. Deletion of Drp1 promotes accumulation of elongated mitochondria in colon cancer cell, leading to the mitochondria-dependent programmed cell death [7]. Targeting inhibition of mitofusin-2 (Mfn-2) and/or Drp1 impairs mitochondrial fission and cell cycle progression, preventing lung cancer cell proliferation [11]. In gastric cancer cells, Drp1-mediated mitochondria events participate in chemoresistance and cell proliferation [12]. Therefore, Drp1-mediated dynamic changes in the mitochondria possibly constitute a promising novel therapeutic target for treating gastric cancer.

Mitochondria harbor a vestige of their original genome (mitochondrial DNAs, mtDNA) and the corresponding full gene expression machinery [13]. Proteins encoded by mtDNA are synthesized in mitochondrial ribosomes [14]. As crucial apoptosis-related factors, mitochondrial ribosomal proteins (RPs) are commonly modified during tumor progression, linking to tumorigenesis and metastasis [^15–17^]. RPs, such as RPS13 and RPL23, have been previously proved as the oncogene in gastric cancer [18]. L22 RP (RPL22), an RP component of the large 60S subunit, is dispensable for both ribosome biogenesis and protein synthesis [19]. Many studies have proved the important regulatory role of RPL22 in various types of cancer including gastric cancer [20,21]. Although the mechanism of Drp1 regulating tumorigenesis has been gradually deepened, few reports unmask the association between Drp1 and RPL22 in gastric cancer.

Herein, we hypothesized that Drp1 integrated an RP to induce the dynamic changes in mitochondria, resulting in the progression of gastric cancer. In the present study, we explored the expression pattern and clinical value of Drp1 in different subtypes of gastric cancer. Furthermore, the molecular function of Drp1 and potential molecular mechanisms by which Drp1-mediated the progression of gastric cancer were deeply explored. Possibly, Drp1 is an effective target for cancer treatment in gastric cancer.

## Materials and methods

### Data collection and analysis

The mRNA expression levels of *drp1* in normal gastric tissues and stomach adenocarcinoma tissues were analyzed using the Gene Expression Profiling Interactive Analysis (GEPIA) version 2 database [22]. The *drp1* mRNA levels in normal gastric tissues and gastric mixed adenocarcinoma tissues, gastric intestinal-type adenocarcinoma tissues and diffuse gastric adenocarcinoma tissues were determined by the dataset downloaded from The Cancer Genome Atlas (TCGA, https://portal.gdc.cancer.gov/). The association between *drp1* expression and *RPL22* was assessed by GEPIA in stomach adenocarcinoma tissues. The association between *drp1* and *RPL22* in subtypes of stomach adenocarcinoma was monitored by SPSS software using the gene expression profiles downloaded from TCGA. RNA interactomes between *drp1* and ribosome pathway and the downstream gene oncogene of *drp1* in ribosome pathway were analyzed using the Encyclopedia of RNA Interactomes (ENCORI) Starbase [23].

### Cell culture and treatment

The immortalized human gastric epithelial cell line GES1 and gastric cancer cell lines including BGC823, MGC803, AGS, and SGC7901 were purchased from Shanghai cell bank, Chinese Academy of Sciences. All cells were cultured with complete Dulbecco’s modified Eagle’s medium (DMEM; 11,320–033, GIBCO, USA) supplemented with 10% fetal bovine serum (10091–148, GIBCO, USA), 100 U/mL penicillin and 100 µg/mL streptomycin (Sigma, USA) in a 37°C humidified incubator with 5% CO_2_. In the inhibitor experiments, BGC823 and SGC7901 cells were pre-treated with 25 μM Mdivi-1 for 12 h [8]. Thereafter, cells were harvested for the subsequent experiments.

### Quantitative real-time PCR (qRT-PCR)

Total RNA was extracted from cultured cells using the Trizol Reagent (Roche, Indianapolis, IN, USA). Reverse transcription was performed using the PrimeScript RT Reagent kit with gDNA Eraser (Takara, Dalian, China) according to the manufacturer’s instructions. For the qRT-PCR analysis, cDNA was amplified using a SYBR Green PCR Kit (Takara, China). The Ct value was collected during the exponential amplification phase. The relative expression level of the target gene was monitored using a 2^−∆∆CT^ method. β-actin was used as internal control. The primers used in this study were listed in **Table S1**.

### Western blot analysis

Cultured cells were dissolved in radioimmunoprecipitation assay lysis buffer containing proteinase inhibitor. Nuclear and cytoplasmic protein extracts of SGC7901 cells were prepared using the Nuclear and Cytoplasmic Extraction Kit (Thermo, 78,833, USA). After determining the protein concentration, 30-μg protein was separated on 10% sodium dodecyl sulfate polyacrylamide gel electrophoresis, and then transferred to polyvinylidene difluoride membrane (Millipore, Bedford, MA). The membranes were subsequently immunoblotted with primary antibodies against Drp1 (1:1000, Abcam, ab184247, UK), Phospho-Drp1^Ser616^ (1:1000, Affinity, AF8470, US) RPL22 (1:2000, Abcam, ab229458, UK), Histone 3 (1:1000, Abcam, ab1791, UK), Bax (1:1000, Abcam, ab263897, UK) and β-actin (1:5000, Sigma, A2228, US). Immunoblots were then visualized with ECL chemiluminescence reagent kit (Millipore, Billerica, MA, USA) according to the manufacturer’s instructions. The intensity of the bands was determined using Quantity One software, and the quantitative analyses of the gray-scale value of each target protein as that of individual β-actin were performed.

### Xenograft model

Five-week-old male nude mice were purchased from the Vital River Laboratory Animal Technology Co. Ltd (Beijing, China) and housed in the Laboratory Animal Center of Xi’an Medical University under controlled temperature (20 ± 2°C) and humidity. All animal studies were approved according to institutional guidelines for laboratory animals. All experiments were approved by the ethics committee of Xi’an Medical University. After a week of adjustable feeding, mice were randomly divided into two groups (five mice/group). Subsequently, approximately 0.2 mL (5 × 10^6^/mL) of SGC7901 cell solution was inoculated subcutaneously into the right flank of mice, allowed to grow for 2 weeks. When tumor volume reached 100 mm^3^ (approximately 2 weeks after the initial injection), Mdivi-1 (1.75 mg/tumor) or equivalent volumes of dimethyl sulfoxide (DMSO, negative control) was injected into each tumor twice a week [11]. Tumor diameters were monitored by measuring two orthogonal dimensional diameters of each tumor thrice weekly. Tumor volume was calculated using the formula: 0.5 × length × width^2^. Three weeks later, the mice were euthanized, and then the dissected tumors were weighed, fixed in 4% paraformaldehyde, or stored at −80°C for the subsequent experiments.

### Tissue microarray and immunohistochemistry

The tissue microarray containing 75 pairs of gastric cancer tissue and the paired para-carcinoma tissue was purchased from OUTDO Biotechnology co. LTD (Shanghai, China). All experiments were approved by the Ethics Committee of Xi’an Medical University. For immunohistochemistry (IHC) assay, the sections (microarray and tumor tissues) were dewaxed in xylene, rehydrated in gradient ethanol, and subjected to antigen retrieval using high-pressure method. The endogenous peroxidase activity was quenched using 3% H_2_O_2_. After blocking with 10% BSA for 1 h, the slices were incubated with primary antibodies against Drp1 (1:200, Abcam, ab184247, UK), Ki67 (1:400, CST, 12,202, US), phospho-histone H3 (1:200, CST, 9701, US), and RPL22 (1:400, Abcam, ab229458, UK) overnight at 4°C. On the following day, the sections were incubated with horse radish peroxidase (HRP)-conjugated secondary antibodies at room temperature for 1 h. Subsequently, the slices were visualized after being stained with DAB (ZL1-9081, ZSGB-BIO, China). Photograph observation was performed under a biological inverted microscope (IX51, Olympus, Japan). Comprehensive analyses included staining intensity, and the number of positive cells was measured using Image Pro Plus 6.0 software (negative: -; weakly positive: +, <20%; middling positive: ++, 20%–50%; strong positive: +++, >50%).

### CCK8 assay

BGC823 and SGC7901 cells were seeded at an initial density of 2000 cells/well in 96-well plates, allowed to grow for 24 h, 48 h, 72 h, and 96 h. Cells were exposed with DMSO or Mdivi-1, respectively. At each time point, all cells were subjected to 10-µL cell counting solution (CCK8, GK10001, GlpBio, US), and the cells were additionally kept for 1 h in a 37°C humidified incubator. Absorbance at 450-nm wavelength was measured using a Spectra Plus microplate reader (Molecular Devices Co., Sunnyvale, CA, USA).

### Apoptosis assay

Briefly, BGC823, and SGC7901 cells were pre-treated with DMSO and Mdivi-1 in 12-well plates. After fusion growth, the digested cells were transferred to centrifuge tube and washed with PBS for twice. Subsequently, cells were stained with Annexin V-FITC Apoptosis Detection Kit (KeyGEN, Nanjing, Jiangsu, China) according to the manufacturer’s protocols. The staining results were detected and analyzed with a flow cytometer (Millipore, USA).

### Tunnel assay

Paraffin sections of tumor tissue in the two groups were assessed using the In Situ Cell Death Detection Kit-POD (Roche, US). Briefly, 5-μm thickness tissue sections were dewaxed in xylene and rehydrated in gradient ethanol. Subsequently, the slices were incubated with DNase-free Proteinase K for 25 min at 37°C and washed thrice with PBS. After 20 min of incubation of cell-penetrating solution, the sections were incubated in situ using Apoptosis Detection Kit (11,684,817,910, Roche, USA) according to the manufacturer’s instructions. Then, the slices were stained by 4’,6-diamidino-2-phenylindole (DAPI) for 10 min in darkness. After mounting, the slices were photographed by fluorescence microscope. The number of TUNEL-positive cells were counted manually and analyzed using Image Pro Plus software.

### Statistical analysis

All experiments were repeated at least thrice. The association between pathological stage and DRP-1 expression in tissue microarray was analyzed using GraphPad Prism 6.0 (GraphPad Software, Inc.) using Fisher’s exact tests. The association between DRP-1 and RPL22 was determined using SPSS software. Kaplan–Meier plotter was used to generate the survival curves using GraphPad Prism, and data were compared between groups using the log-rank (Mantel-Cox) test (univariate Cox regression analysis). Data were presented as the mean ± SD. Statistically significant differences were considered at P < 0.05.

## Results

The present study was aimed to investigate the expression, molecular function, and clinical value of Drp1 in gastric cancer. Mechanistically, Drp1 mediated the nuclear export of RPL22 in gastric cancer cells, leading to the development of gastric cancer. Possibly, Drp1 constitutes a promising novel therapeutic target for treating gastric cancer.

### Drp1 is highly expressed in human gastric carcinoma

**Figure 1.**
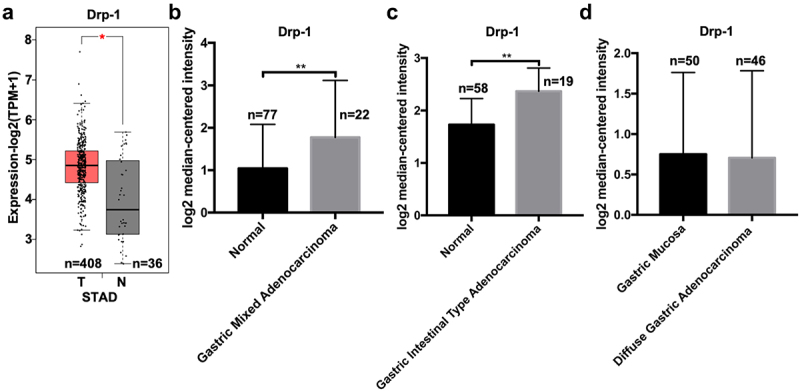
**Expression of Drp1 in human gastric adenocarcinoma**. (a) Expression of Drp1 in human gastric adenocarcinoma and normal tissues was determined by the GEPIA2 database. Differential expression analysis of Drp1 mRNA between normal (n = 77) and gastric mixed adenocarcinoma tissues (n = 22) (b), between normal gastric tissues (n = 58) and gastric intestinal-type adenocarcinoma tissues (n = 19) (c), and between normal gastric mucosa and diffuse gastric adenocarcinoma tissues (n = 46) (d). The data and expression profile were obtained from the TCGA database. *, P < 0.05; **, P < 0.01. T, tumor; N, normal.

**Figure 2.**
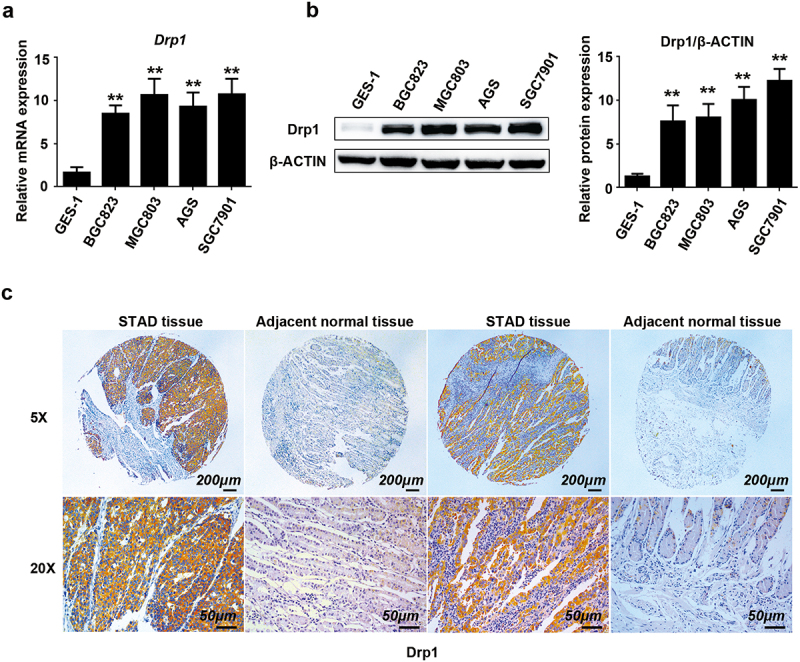
**The expression of Drp1 in gastric cancer cells and tissue microarray**. (a) The expression of *Drp1* mRNA in GES-1, BGC823, MGC803, AGS, and SGC7901 cells as analyzed by quantitative reverse transcription polymerase chain reaction. (b) Drp1 protein levels in GES-1, BGC823, MGC803, AGS, and SGC7901 cells as determined by Western blotting and relative quantification analysis using gray analysis. (c) Representative IHC images of Drp1 expression in the microarray that contained 75 pairs of gastric adenocarcinoma and paired para-carcinoma tissues. Brown color indicates positive staining of Drp1. Scale bar: 200 μm (5x) and 50 μm (20x).

GEPIA2 online analysis was employed to investigate the expression profile of *drp1* mRNA in gastric carcinoma (STAD). The results showed that the expression of *drp1* significantly increased in STAD subjects (n = 408) than that in normal subjects (n = 36) (Figure 1a). Combined with the online Oncomine database, the increased *drp1* mRNA was observed in gastric mixed adenocarcinoma tissue (n = 22) than that in normal gastric tissue (n = 77) (Figure 1b). The mRNA level of *drp1* was also highly expressed in gastric intestinal-type adenocarcinoma tissue (n = 19) than that in normal gastric tissue (n = 58) (Figure 1c). In contrast, there was no significant difference in *drp1* expression between diffuse gastric cancer (n = 46) and normal gastric mucosa (n = 50) (Figure 1d). In addition to the online database analysis, *drp1* mRNA level was highly elevated in the human gastric cancer BGC823, MGC803, AGS, and SGC7901 cells compared with human gastric epithelial cell line GES-1 (Figure 2a). In the translational level, Drp1 protein expression was faintly expressed in human gastric epithelial cell strain GES-1, yet its level was markedly higher in four gastric cancer cell lines (BGC823, MGC803, AGS, SGC7901) (Figure 2b).

Additionally, tissue array that contained 75 pairs of gastric adenocarcinoma and the paired para-carcinoma was employed to further evaluate the expression of *drp1* in STAD tissues. The immunohistochemical staining results demonstrated that the expression of Drp1 protein was prominently elevated in most of the STAD samples compared to that in the adjacent normal tissue (Figure 2c). As shown in Table 1, Drp1 was highly expressed in 66% (48 of 75) of gastric cancer tissues, but only in 33% (25 of 75) of normal tissues (Table 1). In summary, consistent with the online analysis results, these findings present the increased expression of Drp1 in STAD than normal tissue, indicating that Drp1 may be involved in the progression of gastric cancer.

**Table 1.** IHC staining of Drp1 in human stomach adenocarcinoma (STAD) tissues and adjacent normal tissues

Types	Number of patients				P value		
	High expression		Low expression				
Adjacent normal tissues	+++ 3	++ 22	+ 41	-9			
STAD tissues	21	27	21	6			*p* = 0.0001**

-,+,++,+++ represent different degrees of staining.

### Drp1 is significantly associated with clinical features of gastric cancer

**Figure 3.**
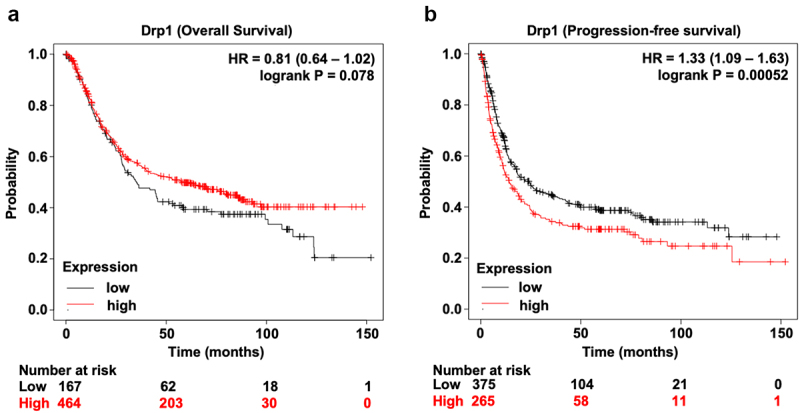
**Prognosis analysis of gastric cancer patients with high and low Drp1**. (a) Probability of overall survival in gastric cancer patients with high and low Drp1. (b) Probability of progression-free survival in gastric cancer patients with high and low Drp1. Black, low Drp1; red, high Drp1.

Next, the correlation between Drp1 expression and clinical features of gastric cancer patients was explored. Among the 75 cases of gastric adenocarcinoma specimens, there were more patients with high Drp1 expression in advanced pathological stages (stage III–IV) (Table 2). Multiple-factor analysis revealed that Drp1 expression had a significant positive correlation with the clinicopathologic stage of gastric cancer patients (P = 0.0484; Table 2). Additionally, the association between Drp1 expression status and the survival probability of gastric cancer patients was also evaluated. As shown in Figure 3a, there was no significant difference in overall survival probability between patients with high *drp1* and those patients with low *drp1* (Figure 3a). However, it was noteworthy that patients with high *drp1* had a lower progression-free survival (PFS: evaluation of drug resistance and curative effect) probability than those with low *drp1* (Figure 3b). Collectively, patients with high Drp1 harbor higher pathological stages and worse PFS probability, implying that Drp1 expression status can be employed as a diagnostic and prognostic hallmark in gastric cancer.

**Table 2.** Correlation between Drp1 expression and pathologic grade of STAD patients

Pathologic grades	Number of patients					P value
	High expression		Low expression			
Stage I Stage II Stage III–IV	+++ 2 9 10	++ 3 10 14	+ 2 14 5	-3 2 1	*p* = 0.0484*

### Drp1 inactivation impairs cell viability and induces apoptosis of gastric cancer cells

**Figure 4.**
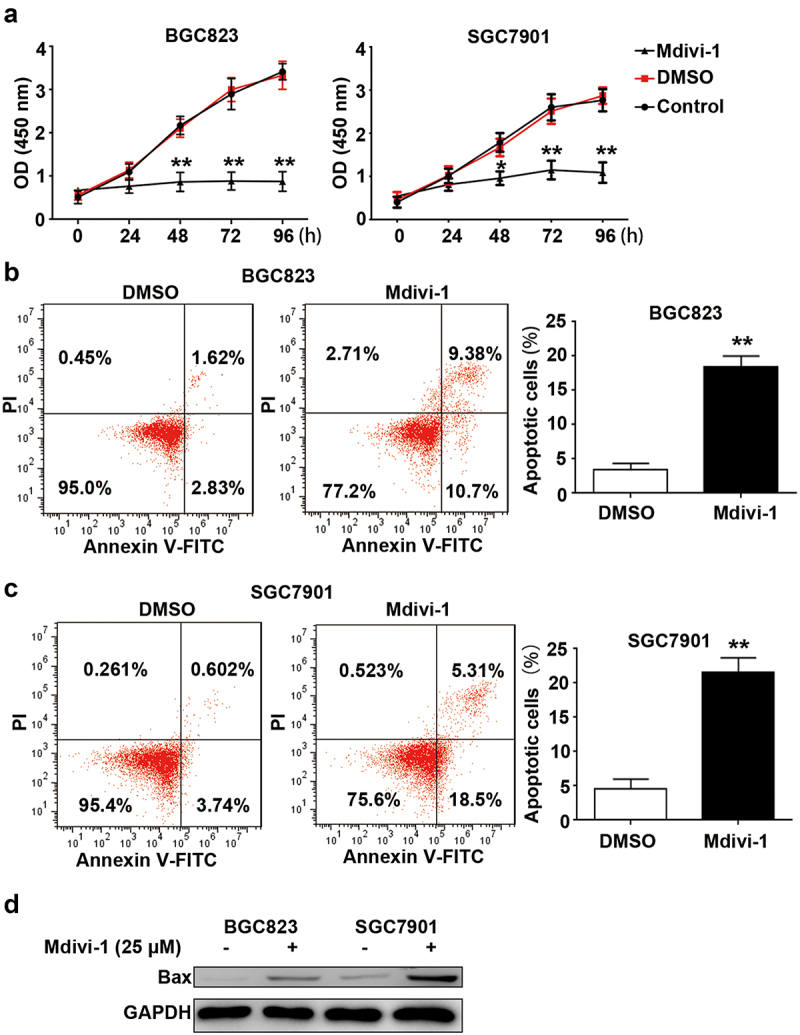
**Effects of Mdivi-1 on proliferation and apoptosis of gastric cancer cells**. (a) BGC823 and SGC7901 cells were treated with DMSO and Mdivi-1. Cell viability was determined by CCK8 assay. (b) After treatment with DMSO and 25 μM Mdivi-1 for 12 h, cell apoptosis was measured using flow cytometry in BGC823 cells. The proportion of apoptotic cells in BGC823 cells exposed DMSO and Mdivi-1 as shown in the right. (c) After treatment with DMSO and Mdivi-1, cell apoptosis was measured using flow cytometry in SGC7901 cells. The proportion of apoptotic cells in SGC7901 cells exposed DMSO and Mdivi-1 as shown in the right. (d) Protein expression of Bax as determined by WB assay in DMSO and Mdivi-1-challenged gastric cancer cells. **, P < 0.01.

To elucidate the role of Drp1 in gastric carcinogenesis, Drp1 GTPase activity was firstly deactivated by Mdivi-1, a selective inhibitor of Drp1. The inhibitor efficiency of Mdivi-1 had been proven by Western blot (WB) in SGC7901 and BGC823 cells. The 12-h Mdivi-1 (25 μM) exposure significantly reduced the Drp1 phosphorylation (Ser 616) in BGC823 and SGC7901 cells (**Fig. S1**). In BGC823 and SGC7901 cells exposed by DMSO, cell viability was increased in a time-dependent manner, while Mdivi-1 exposure significantly decreased cell viability compared to DMSO-treated cells (Figure 4a). There were no differences between control cells and DMSO-exposed cells in cell viability. In addition to cell proliferation, effects of Mdivi-1 on apoptotic events were also assessed. Flow cytometric analysis showed that the proportion of total apoptotic cells (both early and late) was significantly higher in Mdivi-1-exposed BGC823 and SGC7901 cells than that in DMSO-treated cells, increasing by 4.5-fold and 5.5-fold, respectively (Figure 4b-c). Mdivi-1 challenge significantly promoted the expression of Bax in BGC823 and SGC7901 cells (Figure 4d). Taken together, these observations indicate the anti-proliferation effect and pro-apoptosis effect of Drp1 inactivation.

### Drp1 inhibition prevents gastric tumor growth in vivo

**Figure 5.**
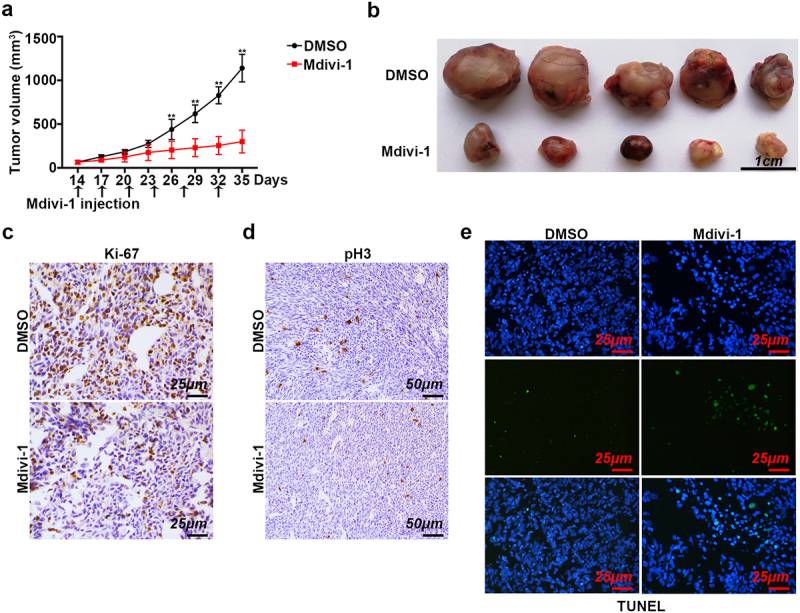
**Role of Drp1 inhibition on tumor growth *in vivo.*** (a) Tumor growth curves of subcutaneous xenograft tumor model developed from SGC7901 cells. Tumor size including tumor length (l) and width (w) was measured using vernier calipers every 3 days in nude mice after 2 weeks of tumor transplantation (day 0). Tumor volumes were calculated according to the formula (L x W x W)/2. (b) Representative excised tumors from animals that were treated either with DMSO or Mdivi-1. Scale bar: 1 cm. (c-d) Representative IHC staining images of Ki-67 and pH3 in xenograft tumors which were treated with DMSO or Mdivi-1. Scale bar: 25 μm and 50 μm. (e) Representative TUNEL staining of xenograft tumors. Tumors obtained from mice exposed with DMSO and Mdivi-1 were subjected to TUNEL for apoptosis detection. Scale bar: 50 μm. Green, apoptotic cells; blue, DAPI. **, P < 0.01.

Xenograft nude mice model was then used to test the effect of Drp1 inactivation on tumor growth *in vivo*. At 2 weeks following the injection of SGC7901 cells, mice were subjected to percutaneous DMSO or Mdivi-1 (1.75 mg/tumor) injection twice a week. After 3 weeks post-treatment, a substantial decrease in tumor volume following treatment with Mdivi-1 was verified, showing a significant decline from day 26 to day 35 in comparison with that in DMSO-challenged mice tumors (Figure 5a). The tumor size was also robustly reduced once treated with Mdivi-1 (Figure 5b). Subsequently, the reduced Ki-67-positive cells and pH3-positive cells were also confirmed in Mdivi-1-treated tumors when compared with those in DMSO-treated tumors (Figure 5c-d). Additionally, TUNEL staining was also performed to determine the late-stage apoptosis events in the two groups. Compared to DMSO group, Mdivi-1 treatment dramatically increased TUNEL-labeled cell signal intensity, presenting more apoptotic cells (Figure 5e). Our data suggest that the blocking of Drp1 activity represses tumor growth and promotes tumor cell apoptosis *in vivo*.

### Drp1 is positively correlated with RPL22 in gastric cancer

**Figure 6.**
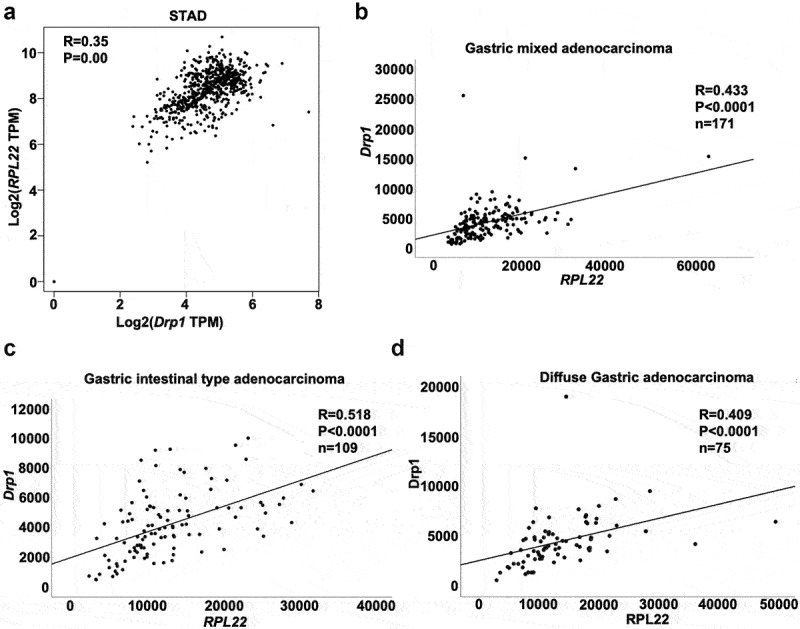
**Association analysis of Drp1 and RPL22 in human gastric adenocarcinoma**. (a) Association between Drp1 and RPL22 in human gastric adenocarcinoma and normal tissues was determined by the GEPIA2 database. Association between Drp1 and RPL22 in gastric mixed adenocarcinoma tissues (b), gastric intestinal-type adenocarcinoma tissues (c), and diffuse gastric adenocarcinoma tissues (d) was analyzed using gene expression profiles downloaded from the TCGA. R, relative coefficient.

Combined with ENCORI Starbase, a significant enriched ‘ribosome pathway’ of Drp1 downstream targets was observed using Kyoto Encyclopedia of Genes and Genomes (KEGG) enrichment analysis. Eighty-eight genes were included in this pathway (Table 3). Using Gene Set Enrichment Analysis, these 88 genes were categorized by gene family. In this study, only RPL22 was verified as a potential downstream oncogene/translocated cancer gene of Drp1 in the ‘ribosome pathway’ using ENCORI Starbase. Additionally, the significant positive association between *drp1* and *RPL22* was observed in STAD samples using GEPIA2 database (R = 0.35; Figure 6a). Of note, in the subtypes of STAD including gastric mixed adenocarcinoma tissues (n = 171) and gastric intestinal-type adenocarcinoma tissues (n = 109) downloaded from the TCGA database, the association between Drp1 and RPL22 was stronger with the higher coefficient (R = 0.433 and R = 0.518; Figure 6b-c). Interestingly, there was also a strong relation between them in diffuse gastric adenocarcinoma tissues (R = 0.409; Figure 6d), implying that Drp1 and RPL22 might be a key signal cascade during the progression of gastric adenocarcinoma.

**Table 3.** RNA interactomes between DNM1L and ribosome pathway

pathwayName	Log10(pval)	Log10(FDR)	backgroundGeneNum	pathwayGeneNum	targetGeneNum	commonGeneNum
KEGG_Ribosome	−3.59352	−1.98074	20,628	88	72	4

### RPL22 may be a crucial downstream oncogene of Drp1 in gastric cancer

**Figure 7.**
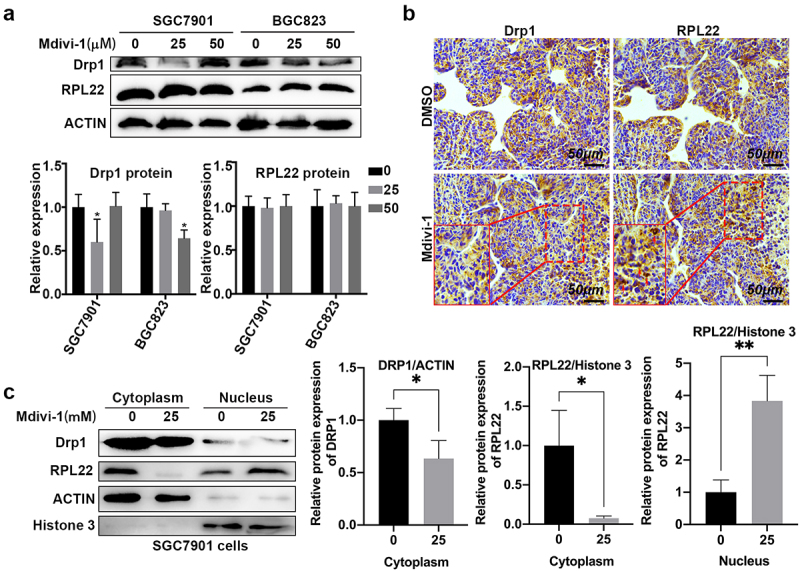
**The association between Drp1 and RPL22**. (a) After treatment with DMSO and Mdivi-1, SGC7901 and BGC823 cells were harvested and protein levels of Drp1 and RPL22 as determined by WB. Relative quantification analysis was conducted using gray analysis (low panel). (b) Xenograft tumors were collected from mice treated with DMSO or Mdivi-1. Protein expression of RPL22 and Drp1 in tumor tissues were determined by IHC assay. Red rectangular frame: the non-overlapping Drp1- and RPL22-positive area; green rectangular frame, the difference of endonuclear location of RPL22 in Mdivi-1-challenged tumor tissues. (c) Nuclear and cytoplasmic protein were prepared and the protein expression of cytoplasmic Drp1 and RPL22 and nuclear Drp1 and RPL22 as determined by Western blotting. *, P < 0.05; **, P < 0.01.

To further reveal the association of RPL22 and Drp1 and RPL22 protein expressions in gastric cancer, cells exposed with DMSO or Mdivi-1 were determined by Western blotting assay. As shown in Figure 7a, 25-μM Mdivi-1 reduced the protein level of Drp1 in SGC7901 cells, while high dose Mdivi-1 (50 μM) did not alter its expression. By contrast, high concentration of Mdivi-1 (50 μM) moderately declined the protein level of Drp1 in BGC823 cells (Figure 7a). However, protein expression of RPL22 did not change in Mdivi-1-exposed SGC7901 and BGC823 cancer cells (Figure 7a). Consistently, no difference in the Drp1 and RPL22 expression was observed in the DMSO- and Mdivi-1-challenged tumors (Figure 7b). Interestingly, the overlapping Drp1- and RPL22-positive area was notably reduced (**red rectangular frame**, Figure 7b), and the intracellular distribution of RPL22 has an endonuclear location in the Mdivi-1-challenged tumor tissues (**green rectangular frame**, Figure 7b). Additionally, in Mdivi-1-challenged SGC7901 cells, Drp1 protein appeared to have a modest decline in the cytoplasm and no changes in the nucleus. However, the cytoplasmic RPL22 was robustly reduced, while nuclear RPL22 notably increased in Mdivi-1-challenged SGC7901 cells (Figure 7c). The data further proved that Mdivi-1-mediated Drp1 inactivation induced RPL22 nuclear translocation. Collectively, RPL22 may be a downstream oncogene of Drp1 and Drp1 inactivation and possibly mediates the nuclear import of RPL22.

However, when we analyzed the expression pattern of RPL22 expression in gastric cancer subjects, no difference was observed between STAD subjects (n = 408) and normal subjects (n = 211) (**Fig. S2A**). Additionally, there was no significant difference in the overall survival probability between patients with high RPL22 and patients with low RPL22 (**Fig. S2B**). Thus, Drp1-mediated development of gastric cancer is associated with nucleo-cytoplasmic transport of RPL22, but not associated with its expression status.

## Discussion

Mitochondrial fission regulation is a therapeutic target in multiple tumor models [24]. Novel approaches targeting mitochondrial fission facilitate good prognosis of malignant tumors via depletion of stem-like tumor cells [25]. In the present study, the increased Drp1 was observed in different subtypes of gastric carcinoma and Drp1 might act as a potential diagnostic and prognostic biomarker of gastric cancer. Inactivation of Drp1 significantly abolished tumor cell proliferation and promoted apoptotic events. As a switch regulator in mitochondrial fission, Drp1-based mitochondrial-targeted therapy could possibly become an effective target for gastric cancer.

Abnormal expression of Drp1 has been verified in diverse cancer types including esophageal squamous cell carcinoma [26] and hematological malignancies [27]. Mitochondrial membrane protein 18 induces the enrichment of Drp1 in mitochondria and subsequently mediates doxorubicin-induced signaling required for mitochondrial fission in gastric cancer cells [12,28]. In this study, the accumulation of Drp1 in gastric cancer tissues and cancer cells was observed. However, in diffuse gastric adenocarcinoma, no changes were observed in Drp1 expression compared to that in gastric mucosa tissues. Therefore, Drp1 may exhibit molecular heterogeneity characteristic of different subtypes of gastric adenocarcinoma. Based on Oncomine and TCGA databases, a significant negative correlation between Drp1 expression status and overall survival (OS) of lung cancer patients was discovered [29]. Patients with a high Drp1^Ser616^ activity are associated with a high risk of developing tumor relapse, poor 5-year disease-free survival, and 5-year OS after neoadjuvant chemoradiotherapy therapy in locally advanced rectal cancer [30]. Here, no association between Drp1 and OS was verified in gastric cancer cohorts. However, the numbers at risk in the high Drp1 group were more than that in the low DRP1 group, indicating that patients with high Drp1 were at high risk of death. The significant variation of PFS between high Drp1 and low Drp1 groups suggested that Drp1 expression was negatively associated with outcomes or resistance of gastric cancer patients after drug intervention. Whether Drp1 can be used as a vital predictor for distinguishing the prognosis of gastric cancer patients treated with different drugs, such as chemotherapy, targeted drugs, or immunotherapy, remains unclear.

Dysregulated activity of Drp1 GTPase causes disrupted mitochondrial dynamics and cellular metabolism, facilitating cancer cell survival and proliferation [31]. The current standard Drp1 GTPase inhibitor, Mdivi-1, obviously inhibits fissogenic activity, resulting in the reduction of cell proliferation and tumor growth and the promotion of cell apoptosis in multiple cancer cells [32]. Additionally, Mdivi-1 administration also sensitizes cholangiocarcinoma cells to the cytotoxicity of cisplatin through cellular apoptosis induction via the mitochondrial pathway [33]. In gastric cancer cells, the Mdivi-1-mediated the inactivation of Drp1 GTPase also impeded cell proliferation and tumor growth and induced apoptotic events. Therefore, disruption of mitochondrial dynamics using Drp1 GTPase inhibitor may be a novel strategy to improve the therapeutic strategy for treating gastric cancer. Besides, the Mdivi-1-mediated decrease in oxidative metabolism and the disruption in the M phase cell cycle progression induce the impairment on cancer cell proliferation [34,35]. Possibly, the Mdivi-1-induced cell apoptosis was attributed to oxidative metabolism disorders and cell cycle progression. Mdivi-1 significantly reduced the expression of Drp1 and phosphorylated Drp1^Ser616^, attenuating the release of cytochrome C from the mitochondria [36]. We also verified the moderate decline in the Drp1 protein level along with the inactivation of Drp1 in the Mdivi-1-exposed gastric cancer cells, but there are no consecutive effects in tumor tissues possibly due to the influence of the tumor microenvironment or other reasons. Actually, Mdivi-1 mainly mediated the decreased activity of Drp1 rather than its expression in the cell and animal models. Although Mdivi-1 reduced the protein expression of Drp1 in SGC7901 cells, it could not prove the role of Drp1 protein expression in the development of gastric cancer. To further explore the role of Drp1 in gastric cancer progression, a Drp1-knockout and overexpression experiments in gastric cancer cells for assessing tumor behaviors are warranted.

The dysfunction of the RPL22 can induce cancer risk. The binding of RPL22 to casein kinase 2α inhibits substrate phosphorylation of casein kinase 2α, resulting in lung cancer cell apoptosis [37,38]. Combined with the ENCORI Starbase and Western blot analysis, RPL22 in the ribosome pathway was a candidate downstream oncogene of Drp1. A significant positive association between Drp1 and RPL22 in different subtypes of gastric carcinoma was confirmed. Thus, the implication of Drp1 in the ribosome pathway may be associated with RPL22 in gastric cancer cells. Commonly, RPL22 is located in the cytoplasm [39]. In our data, the overlaps of Drp1 and RPL22 expression was observed in the cytoplasm of gastric cancer tumors. Probably, the accumulation of Drp1-enriched RPL22 in the cytoplasm resulted in the disorder of mitochondrial dynamics, inducing over-proliferation of tumor cells. Human mitochondrial ribosome (mitoribosome) is composed of a small 28-S subunit, which includes 12S rRNA and 29 proteins and a large 39-S subunit, which includes 16S rRNA and 48 proteins, and all these ribosomal proteins are encoded in the nucleus and subsequently imported into the mitochondrial matrix [40]. Among the 48 distinct proteins of human mitochondrial 39-S subunit, one has the homologs of the 50-S ribosomal protein L22, implying that RPL22 homologs exist in the mitochondria [41]. Mitochondrial ribosomal proteins are key components for protein synthesis encoded by mtDNA and mitochondrial homeostasis. In addition, to control mitoribosome assembly, mitochondrial ribosome proteins also participate in cellular regulation like cell cycle, apoptosis, and mitochondrial dynamics [42]. Based on the overlaps of Drp1 and RPL22 in the cytoplasm, we speculated that the homologs of RPL22 in the mitochondria might participate in Drp1-mediated dysfunction of mitochondrial dynamics and cell proliferation in gastric cancer. Of note, as an RNA-binding ribosomal protein (RP), cytoplasmic RPL22 can transfer to the nucleus, contributing to ribosome biogenesis and protein synthesis, influencing ‘extraribosomal’ cellular processes via binding to the downstream mRNAs [20]. In the present study, the nuclear import of RPL22 from the cytoplasm was observed in Drp1-inactivated tumors. Possibly, the increased nuclear RPL22 may bind to the downstream tumor suppressor genes in the nucleus, exerting an effective anti-tumor role. However, our data only provided some association between Drp1 and RPL22. It is the lack of a more putative evidence to confirm the nuclear import of RPL22 and its function in Drp1-inactivated gastric cancer cells. Although the Mdivi-1-mediated inactivation of Drp1 or the decline of Drp1 protein level did not affect the protein expression of RPL22, more experiments such as the knockdown of Drp1 or the nuclear transport prevention approach should be conducted to reveal the Drp1/RPL22 signaling axis in Mdivi-1-challenged gastric cancer cells.

## Conclusion

In summary, Drp1 was elevated in different gastric cancer subtypes except for diffuse gastric adenocarcinoma. Drp1 had a potential diagnostic and prognostic value for tumor stage and PFS rate of gastric adenocarcinoma patients, respectively. The inactivation of Drp1 facilitated cell apoptosis and inhibited the proliferation and tumor growth of gastric cancer cells, which was possibly associated with the nuclear import of RPL22. Possibly, the regulation of mitochondrial fission via the Drp1 inactivation was a promising novel therapeutic strategy for gastric cancer.

## Supplementary Material

Supplemental Material

## Abbreviations

Drp1, Dynamin-related protein 1; RPL22, L22 ribosomal protein; GEPIA2, Gene Expression Profiling Interactive Analysis 2; ENCORI, Encyclopedia of RNA Interactomes; ROS, reactive oxygen species; GTPases, guanosine triphosphatases; Mfn-2, mitofusin-2; mtDNA, mitochondrial DNAs; TCGA, The Cancer Genome Atlas.
