# Supplementary material for: L22 ribosomal protein is involved in dynamin-related protein 1-mediated gastric carcinoma progression
Source: Bioengineered. 2022 Mar 1;13(3):6650–64. doi: 10.1080/21655979.2022.2045842 (PMC9208493; doi:10.1080/21655979.2022.2045842)
Supplement: Supplemental Material [file KBIE_A_2045842_SM6358.zip › supplementary/Table S1.docx]

**Table S1. Primer sequences of qRT-PCR.**

| **Gene** | Forward | Reverse |
| --- | --- | --- |
| ***Drp1*** | 5’-GGAGACTCATCTTTGGTGAAGAG-3’ | 5’-AAGGAGCCAGTCAAATTATTGC-3’ |
| ***β-actin*** | 5’-CACGATGGAGGGGCCGGACTCATC-3’ | 5’-TAAAGACCTCTATGCCAACACAGT-3’ |
