# Supplementary figures and images for: L22 ribosomal protein is involved in dynamin-related protein 1-mediated gastric carcinoma progression
Source: Bioengineered. 2022 Mar 1;13(3):6650–64. doi: 10.1080/21655979.2022.2045842 (PMC9208493; doi:10.1080/21655979.2022.2045842)

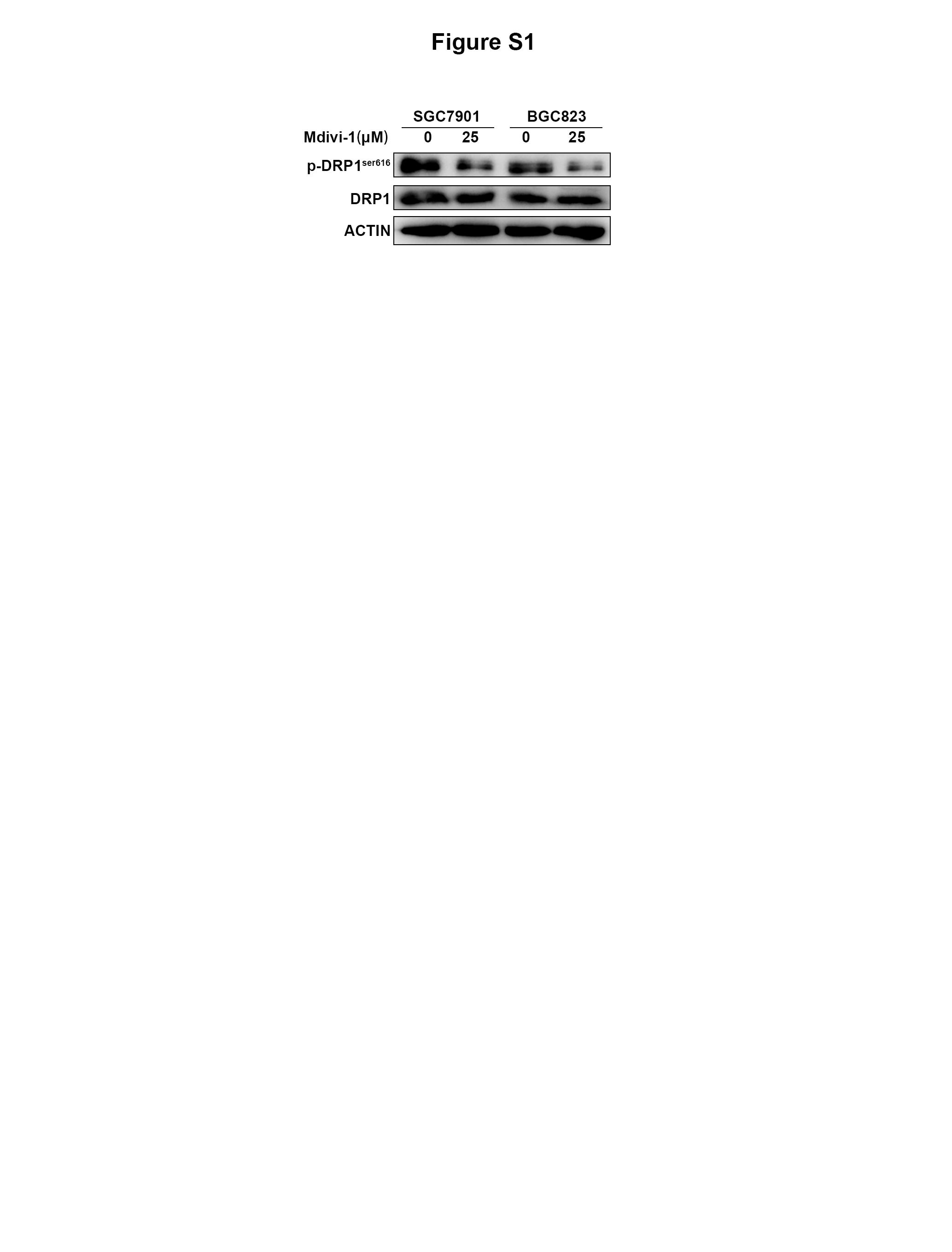

Supplement: Supplemental Material [file KBIE_A_2045842_SM6358.zip › supplementary/Fig S1.tif]

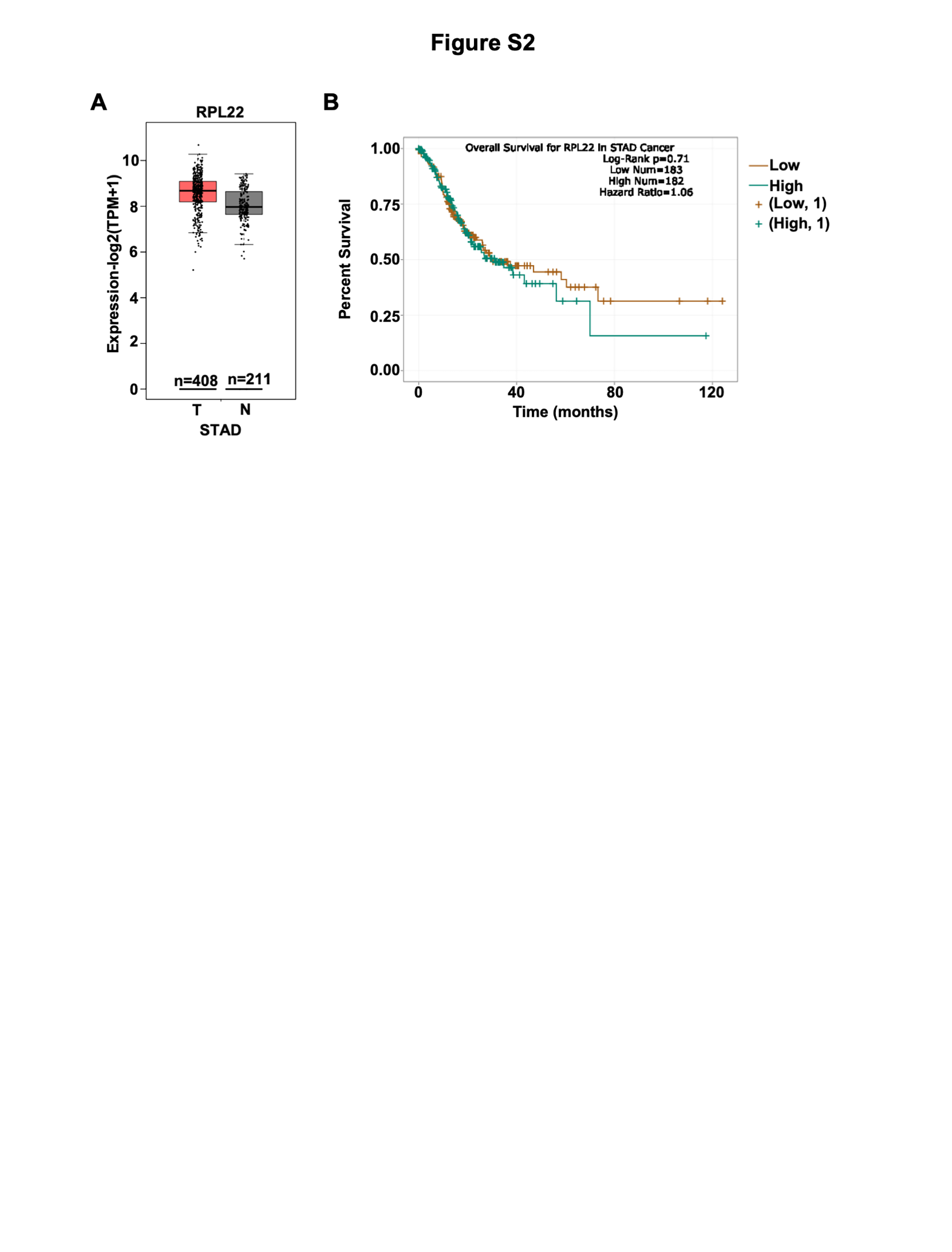

Supplement: Supplemental Material [file KBIE_A_2045842_SM6358.zip › supplementary/Fig S2.tif]
